# Enhancing gastroenterology with multimodal learning: the role of large language model chatbots in digestive endoscopy

**DOI:** 10.3389/fmed.2025.1583514

**Published:** 2025-05-21

**Authors:** Yuanyuan Qin, Jianming Chang, Li Li, Mianhua Wu

**Affiliations:** ^1^First Clinical Medical College, Nanjing University of Chinese Medicine, Nanjing, China; ^2^Jiangsu Collaborative Innovation Center of Traditional Chinese Medicine Prevention and Treatment of Tumor, Nanjing University of Chinese Medicine, Nanjing, China; ^3^School of Computer Science and Engineering, Southeast University, Nanjing, China

**Keywords:** multimodal learning, large language models, digestive endoscopy, AI-assisted diagnosis, domain adaptation

## Abstract

**Introduction:**

Advancements in artificial intelligence (AI) and large language models (LLMs) have the potential to revolutionize digestive endoscopy by enhancing diagnostic accuracy, improving procedural efficiency, and supporting clinical decision-making. Traditional AI-assisted endoscopic systems often rely on single-modal image analysis, which lacks contextual understanding and adaptability to complex gastrointestinal (GI) conditions. Moreover, existing methods struggle with domain shifts, data heterogeneity, and interpretability, limiting their clinical applicability.

**Methods:**

To address these challenges, we propose a multimodal learning framework that integrates LLM-powered chatbots with endoscopic imaging and patient-specific medical data. Our approach employs self-supervised learning to extract clinically relevant patterns from heterogeneous sources, enabling real-time guidance and AI-assisted report generation. We introduce a domain-adaptive learning strategy to enhance model generalization across diverse patient populations and imaging conditions.

**Results and discussion:**

Experimental results on multiple GI datasets demonstrate that our method significantly improves lesion detection, reduces diagnostic variability, and enhances physician-AI collaboration. This study highlights the potential of multimodal LLM-based systems in advancing gastroenterology by providing interpretable, context-aware, and adaptable AI support in digestive endoscopy.

## 1 Introduction

Gastroenterology has witnessed significant advancements with the integration of artificial intelligence (AI), particularly in digestive endoscopy, where precise diagnosis, decision support, and workflow optimization are critical (1). Traditional endoscopic assessments rely heavily on expert interpretation, which can be time-consuming, subject to inter-operator variability, and prone to misdiagnosis. Multimodal learning, which combines visual, textual, and real-time patient data, has emerged as a promising approach to enhance endoscopic decision-making (2). Large Language Model (LLM)-based chatbots are at the forefront of this transformation, providing real-time guidance, differential diagnosis suggestions, and automated report generation by synthesizing multiple sources of information (3). Not only do these AI-driven tools reduce the cognitive load on physicians, but they also enable standardization in endoscopic interpretations and improve diagnostic accuracy. Integrating multimodal AI in gastroenterology allows for more efficient data-driven decision-making by leveraging real-time endoscopic imagery, electronic health records, and clinical guidelines (4). Despite these benefits, current AI solutions still face challenges related to interpretability, real-time responsiveness, and clinical integration. To address these limitations, researchers have explored various approaches, evolving from traditional knowledge-based systems to data-driven machine learning techniques and, more recently, deep learning and large pre-trained models. This paper reviews the progression of these techniques and discusses their impact on digestive endoscopy (5).

Early approaches to AI-assisted digestive endoscopy primarily relied on symbolic reasoning and knowledge-based systems. These rule-based systems utilized predefined expert knowledge and ontologies to analyze endoscopic findings and recommend possible diagnoses (6). For example, early expert systems integrated structured image descriptors with endoscopic procedural guidelines to identify abnormalities such as ulcers, polyps, and malignancies. Similarly, ontology-driven frameworks enabled AI tools to standardize reporting by mapping visual findings to structured diagnostic terms (7). While these systems provided interpretability and consistency, they suffered from limited adaptability to new endoscopic techniques and variations in imaging conditions. The reliance on handcrafted rules restricted their ability to generalize across diverse patient populations and evolving clinical knowledge (8). The static nature of these systems made it challenging to incorporate continuous learning from new data, limiting their effectiveness in real-world endoscopic practice. To overcome these drawbacks, researchers shifted toward data-driven machine learning approaches, which offered improved flexibility and learning capabilities (9).

The advent of machine learning models revolutionized AI-assisted digestive endoscopy by facilitating automated pattern recognition from extensive endoscopic datasets. Techniques such as support vector machines (SVMs), random forests, and convolutional neural networks (CNNs) were employed to classify endoscopic images and detect lesions with greater accuracy (10). For instance, machine learning-based image segmentation allowed automated detection of polyps and early-stage cancers, reducing the need for manual annotation. Probabilistic models improved endoscopic decision support by analyzing multimodal patient data, including clinical history and histopathological reports (11). Despite their improved adaptability compared to rule-based systems, traditional machine learning methods required extensive feature engineering and manual tuning to optimize performance. Moreover, these models struggled with real-time inference in endoscopic procedures due to computational constraints (12). Another limitation was the lack of contextual understanding, as machine learning models primarily focused on single-modality data, such as images or structured patient records, without integrating textual and conversational aspects. The advent of deep learning and large pre-trained language models provided a solution to these challenges (13).

Deep learning and multimodal learning techniques have greatly propelled AI-driven innovations in digestive endoscopy, allowing for automatic feature extraction and seamless real-time integration of multimodal data. Large-scale CNNs and transformer-based architectures have demonstrated exceptional performance in analyzing endoscopic videos, detecting abnormalities, and providing diagnostic predictions with high accuracy (14). More recently, Large Language Model (LLM)-driven chatbots have revolutionized AI-assisted gastroenterology by facilitating real-time interaction between physicians and AI systems. These chatbots integrate multimodal learning by combining visual endoscopic findings with clinical text-based insights, enhancing decision support (15). For example, an LLM-powered chatbot can analyze endoscopic images, retrieve relevant clinical literature, and suggest differential diagnoses in real time, assisting gastroenterologists in complex cases. Transformer-based architectures enable dynamic adaptation to evolving medical knowledge by continuously learning from new datasets (16). However, challenges remain in terms of interpretability, potential biases in training data, and real-time deployment in high-stakes clinical settings. Addressing these issues requires advancements in explainable AI and real-time processing frameworks (17).

Building on these developments, we propose a novel framework that leverages multimodal learning and LLM-driven chatbots to enhance digestive endoscopy. Our approach integrates transformer-based AI models with real-time endoscopic imaging and structured clinical knowledge to provide interactive decision support. Unlike traditional symbolic AI, our framework is not restricted by static rules and can dynamically adapt to new endoscopic techniques and imaging modalities. It surpasses conventional machine learning methods by incorporating multimodal fusion, enabling a more comprehensive understanding of patient conditions. To improve clinical trustworthiness, our approach incorporates explainable AI mechanisms, ensuring that endoscopic findings and chatbot-generated recommendations are transparent and interpretable. By leveraging pre-trained language models and real-time data processing, our method enhances diagnostic accuracy, procedural efficiency, and physician-AI interaction in gastroenterology.

The proposed method has several key advantages:

- Our framework introduces a transformer-based multimodal learning approach that integrates endoscopic imaging, clinical reports, and LLM-driven chatbots to enhance diagnostic accuracy and procedural decision-making.
- Unlike conventional machine learning models, our method processes multimodal data in real-time, providing interactive decision support for gastroenterologists, improving workflow efficiency in digestive endoscopy.
- Experimental evaluations demonstrate that our approach outperforms existing AI-assisted endoscopy methods in accuracy, adaptability, and physician usability, ensuring seamless clinical integration and improved patient outcomes.

## 2 Related work

### 2.1 Multimodal learning for enhanced gastrointestinal diagnostics

Multimodal learning has emerged as a transformative approach in gastroenterology, integrating various data sources such as endoscopic imaging, clinical records, and genetic information to improve diagnostic accuracy and patient management (18). By leveraging multimodal data, AI-driven models can provide comprehensive insights into gastrointestinal conditions, aiding in both early detection and treatment planning. A prominent example is the application of multimodal AI in diagnosing pancreatic lesions. A randomized crossover study demonstrated that combining endoscopic ultrasound images with patient-specific clinical data resulted in superior diagnostic performance compared to conventional single-modal approaches (19). This highlights the value of integrating multiple data types to enhance clinical decision-making. Multimodal machine learning models have also shown promise in endoscopy by improving the detection and characterization of gastrointestinal abnormalities (20). By synthesizing visual endoscopic data with patient history and histopathological reports, these models enable real-time, highly accurate assessments, assisting endoscopists in making informed decisions during procedures (21). The integration of advanced diagnostic tools, such as white-light endoscopy combined with confocal laser endomicroscopy, has facilitated real-time *in vivo* histological assessment of tissues (22). This approach has significantly improved the detection of conditions such as Barrett's esophagus and other precancerous lesions, demonstrating the potential of multimodal AI in gastroenterology (23).

### 2.2 The role of large language models in digestive endoscopy

Large Language Models (LLMs) have introduced new possibilities in digestive endoscopy, particularly in areas such as patient education, clinical decision support, and AI-assisted report generation. These AI-driven chatbots can process and generate human-like text, making them highly valuable tools in modern gastroenterological practice (24). One key application of LLMs is personalized patient education. AI-driven chatbots can provide tailored information regarding upcoming endoscopic procedures, post-procedure care, and common patient concerns. This personalized approach not only enhances patient comprehension but also increases overall satisfaction with medical procedures (25). In diagnostic applications, integrating LLMs with multimodal AI models has proven effective in assessing complex gastrointestinal conditions. For instance, deep-learning systems trained on combined white-light and weak-magnifying endoscopic images have demonstrated real-time diagnostic capabilities (26), accurately identifying neoplastic lesions and aiding endoscopists during procedures. Beyond diagnostics, LLM chatbots have shown potential in clinical decision support by synthesizing multimodal data—such as endoscopic imaging, histopathological findings, and electronic health records—to provide tailored treatment recommendations (27). By analyzing a patient's medical history and current symptoms, these chatbots can suggest treatment options, dietary modifications, and follow-up schedules, improving adherence to medical advice and personalized patient care (28). LLMs facilitate advanced training for healthcare professionals by simulating complex clinical scenarios that incorporate diverse data types. These AI-driven simulations enhance diagnostic reasoning and decision-making skills, making them valuable tools in medical education (29).

Recent clinical studies have begun to demonstrate the measurable benefits of LLM-based assistance in real-world medical workflows. For instance, Pellegrino et al. (21) conducted a concordance analysis in colonoscopy, showing that ChatGPT-4-assisted scoring of bowel preparation quality achieved comparable results to expert gastroenterologists, while improving documentation consistency and reducing inter-rater variability (28). Similarly, Chai and Wang reported that LLM-powered clinical decision support systems, when integrated with EHR data, improved diagnostic agreement rates in complex gastrointestinal cases by over 12% compared to standard rule-based systems (27). These findings support the claim that LLMs can effectively augment physician decision-making and documentation processes, especially when applied in structured, supervised clinical settings.

### 2.3 Challenges and future directions in AI-driven gastroenterology

Despite the significant advancements in AI-driven gastroenterology, challenges remain in integrating multimodal learning and LLMs into clinical practice. Ensuring interoperability between diverse data sources, maintaining patient privacy (30), and achieving high clinical accuracy are key obstacles that require further research and development. One of the primary challenges is the variability in data sources and imaging techniques across different institutions. AI models must be trained to handle domain shifts and variations in endoscopic imaging conditions to ensure reliable and consistent performance (31). Domain-adaptive learning strategies have been proposed to improve generalization, but further validation is needed for widespread clinical adoption. Another critical challenge is the interpretability and transparency of AI-driven decision support systems (32). While deep learning models offer superior accuracy, their black-box nature poses difficulties in clinical acceptance. Explainable AI (XAI) techniques are crucial for increasing trust among clinicians by providing insights into how AI models generate their recommendations (33). Real-time deployment of AI models in high-stakes clinical settings presents computational challenges. Multimodal learning frameworks require substantial processing power to analyze large-scale endoscopic video data alongside patient-specific records (34). Advancements in model efficiency, including optimization techniques such as quantization and pruning, are necessary to facilitate seamless integration into real-world healthcare workflows. Ethical considerations surrounding AI applications in gastroenterology must be addressed (35). Ensuring unbiased training datasets, preserving patient confidentiality, and adhering to regulatory frameworks are essential to the responsible deployment of AI in medical practice. Collaborative efforts among clinicians, AI researchers, and regulatory bodies are critical for overcoming these challenges and fully realizing the potential of AI in digestive endoscopy (36).

## 3 Method

### 3.1 Overview

Artificial Intelligence (AI) has emerged as a transformative technology in gastroenterology, enhancing diagnostic accuracy, optimizing treatment strategies, and improving patient outcomes. With the increasing complexity and volume of medical data, AI-driven approaches offer new possibilities for automating image interpretation, predicting disease progression, and personalizing patient care. This section provides an overview of our proposed methodology, which integrates AI models into gastroenterology workflows, covering key components such as problem formulation, model development, and novel optimization strategies.

In Section 3.2, we present the preliminaries necessary to understand the application of AI in gastroenterology. This includes defining the imaging modalities commonly used in gastrointestinal (GI) diagnostics, such as endoscopy, radiology, and histopathology, and formulating the AI-driven decision-making process. Key mathematical representations of data acquisition, preprocessing, and feature extraction are introduced to establish a structured foundation for AI integration. In Section 3.3, we introduce our novel AI-based model tailored for gastroenterology. Unlike conventional rule-based or handcrafted feature extraction methods, our approach employs deep learning architectures to automatically learn discriminative features from GI images and clinical data. By incorporating self-supervised learning and multi-modal data fusion, our model achieves robust performance across diverse patient populations and varying imaging conditions. In Section 3.4, we propose a new strategy to optimize AI deployment in clinical settings. This involves designing interpretable AI systems that provide explainable decision support for gastroenterologists. We introduce a domain-adaptive learning technique to enhance model generalization, mitigating biases associated with dataset variations. The proposed strategy also includes an uncertainty quantification mechanism to assist clinicians in assessing model confidence and reliability. We systematically develop a comprehensive AI framework for gastroenterology, leveraging state-of-the-art machine learning techniques to advance disease detection, risk assessment, and therapeutic planning.

**Figure 1:**
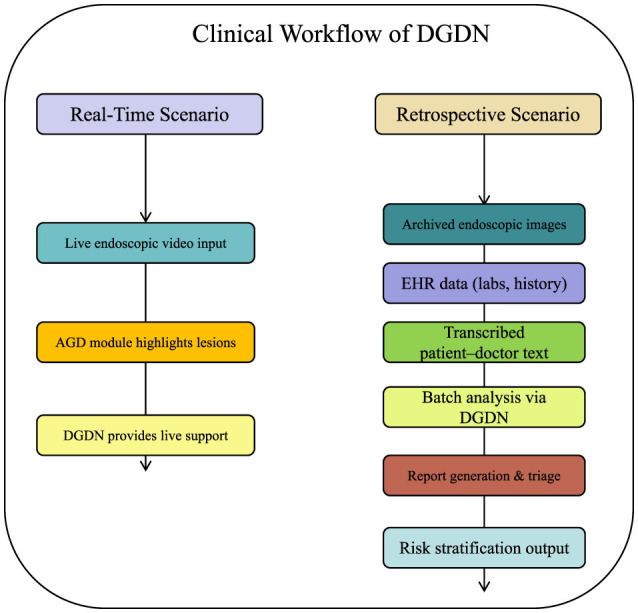
Illustration of DGDN in clinical workflow. **Left**: real-time support during live endoscopy. **Right**: retrospective analysis for diagnostic reporting and triage. The multimodal AI model integrates live image input, electronic health records, and clinical dialogue to provide context-aware, interpretable decision support at multiple stages of patient care.

To further contextualize the application of our proposed framework, we illustrate how DGDN can be deployed within real-world clinical workflows. DGDN is designed to support both real-time diagnostic assistance during endoscopic procedures and retrospective decision support for clinical reporting and triage. In a real-time scenario, the DGDN model ingests endoscopic video frames on-the-fly, applies the AGD module to highlight diagnostically relevant regions, and generates live predictions with uncertainty quantification. This assists gastroenterologists in identifying suspicious lesions, guiding biopsy decisions, or confirming visual impressions during procedures. Alternatively, in a retrospective setting, the model processes archived endoscopic images, structured clinical records, and transcribed doctor–patient dialogue from electronic health systems. By fusing these multimodal inputs, DGDN can generate structured diagnostic summaries, suggest follow-up actions, or prioritize cases based on risk levels. This supports applications such as endoscopy reporting automation, post-procedure quality assurance, and early-stage triage. A schematic diagram of this workflow is shown in Figure 1, highlighting the flexibility of DGDN in adapting to various points of care in gastroenterology.

### 3.2 Preliminaries

The application of Artificial Intelligence (AI) in gastroenterology primarily focuses on analyzing medical imaging data, automating disease detection, and enhancing clinical decision-making. To formalize this problem mathematically, we define the structure of AI-assisted gastroenterological diagnostics through a rigorous formulation of the data, feature space, and inference mechanism.

Medical imaging plays a central role in gastroenterology, encompassing modalities such as endoscopy, computed tomography (CT), magnetic resonance imaging (MRI), and histopathological slides. Each imaging modality provides a different data structure, which we define as follows.

Given an imaging modality *m*, let $I_{m}$ represent the space of all possible images captured using this modality. An image sample is then denoted as:


(1) $$\begin{array}{l} \text{X}\in I_{m},\;\text{X}=\left\{x_{i,j,c}|i\in \left[1,H\right],j\in \left[1,W\right],c\in \left[1,C\right]\right\}, \end{array}$$

where *H* and *W* are the height and width of the image, and *C* is the number of channels.

Each image **X** is associated with a diagnostic label y∈Y, where Y represents the set of possible conditions. The goal of AI-based diagnosis is to learn a function $f:I_{m}\to Y$ that accurately maps an input image to its corresponding diagnosis.

To enable effective AI modeling, we define a feature space F that captures relevant patterns in gastrointestinal imaging. A feature vector **z** extracted from an image **X** is defined as:


(2) $$\begin{array}{l} \text{z}=\phi \left(\text{X};\theta \right), \end{array}$$

where ϕ(·;θ) is a feature extraction function parameterized by θ, typically learned using deep neural networks.

In the case of endoscopy images, features may include textural patterns, lesion boundaries, and color variations, while in histopathological images, cellular morphology and tissue organization are key factors. The extracted features **z**∈ℝ^*d*^ form a high-dimensional representation that serves as input to classification or segmentation models.

Given an image **X**, the AI model predicts the likelihood of different conditions by computing:


(3) $$\begin{array}{l} p\left(y|\text{X}\right)=g\left(\text{z};\theta_{g}\right), \end{array}$$

where *g*(·;θ_*g*_) is a classification function, typically modeled as a neural network with softmax output:


(4) $$\begin{array}{l} p\left(y_{k}|\text{X}\right)=\frac{\exp\left({\text{w}}_{k}^{⊤}\text{z}+b_{k}\right)}{\sum_{j=1}^{\left|Y\right|}\exp\left({\text{w}}_{j}^{⊤}\text{z}+b_{j}\right)}, \end{array}$$

where **w**_*k*_ and *b*_*k*_ are the parameters corresponding to class *k*. The predicted class ŷ is then given by:


(5) $$\begin{array}{l} ŷ=\arg\underset{y_{k}\in Y}{\max}p\left(y_{k}|\text{X}\right). \end{array}$$

In real-world gastroenterology applications, data often exhibits spatial and temporal dependencies. For example, an endoscopy video provides sequential frames ${\left\{{\text{X}}_{t}\right\}}_{t=1}^{T}$ capturing dynamic views of the gastrointestinal tract. A temporal AI model can be formulated as:


(6) $$\begin{array}{l} {\text{z}}_{t}=\phi \left({\text{X}}_{t};\theta \right), \end{array}$$

(7) $$\begin{array}{l} {\text{h}}_{t}=\psi \left({\text{z}}_{t},{\text{h}}_{t-1};\theta_{\psi }\right), \end{array}$$

where ψ represents a recurrent function that accumulates past information through a hidden state **h**_*t*_.

Beyond imaging, gastroenterology AI systems can leverage multi-modal data, including patient history, laboratory test results, and genetic profiles. Given *N* data modalities {*m*_1_, *m*_2_, ..., *m*_*N*_}, each providing a feature set **z**^(*m*)^, a fused representation is obtained via:


(8) $$\begin{array}{l} {\text{z}}_{\text{fusion}}=\Omega \left({\text{z}}^{\left(m_{1}\right)},{\text{z}}^{\left(m_{2}\right)},...,{\text{z}}^{\left(m_{N}\right)}\right), \end{array}$$

where Ω(·) is a fusion function, which may include concatenation, attention mechanisms, or graph-based integration.

A major challenge in AI-based gastroenterology is ensuring robustness across diverse imaging conditions and patient populations. A domain adaptation approach can be formulated as:


(9) $$\begin{array}{l} L_{\text{adapt}}=E_{\text{X}\sim D_{s}}L\left(f\left(\text{X}\right),y\right)+\lambda D\left(F_{s},F_{t}\right), \end{array}$$

where $D_{s}$ and $D_{t}$ are the source and target domain distributions, and *D*(·, ·) measures the feature space discrepancy, often implemented using Maximum Mean Discrepancy (MMD) or adversarial alignment.

### 3.3 Deep gastrointestinal diagnosis network

**Figure 2:**
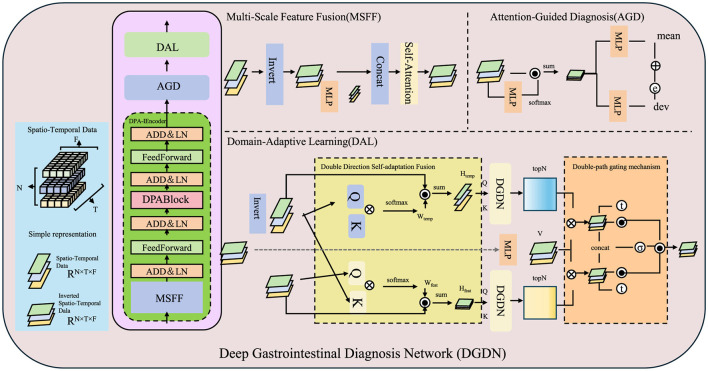
Overview of the Deep Gastrointestinal Diagnosis Network (DGDN). The architecture consists of three key components, Multi-Scale Feature Fusion (MSFF), Attention-Guided Diagnosis (AGD), and Domain-Adaptive Learning (DAL). MSFF extracts and integrates multi-scale spatial features to capture both fine-grained pathological details and global contextual patterns. AGD employs an adaptive attention mechanism to enhance diagnostically relevant regions while suppressing background noise. DAL ensures robust feature generalization across different imaging domains through adversarial domain adaptation and contrastive learning. The model leverages spatio-temporal data representation, hierarchical feature extraction, and a self-adaptive fusion mechanism to improve disease detection, segmentation, and classification in gastrointestinal imaging.

To address the challenges in AI-assisted gastroenterology, we propose the Deep Gastrointestinal Diagnosis Network (DGDN), a novel deep learning architecture designed to improve disease detection, segmentation, and classification in gastrointestinal (GI) imaging. Unlike traditional models, DGDN integrates multiple learning paradigms to enhance diagnostic accuracy and generalization (As shown in Figure 2).

#### 3.3.1 Multi-scale feature fusion

**Figure 3:**
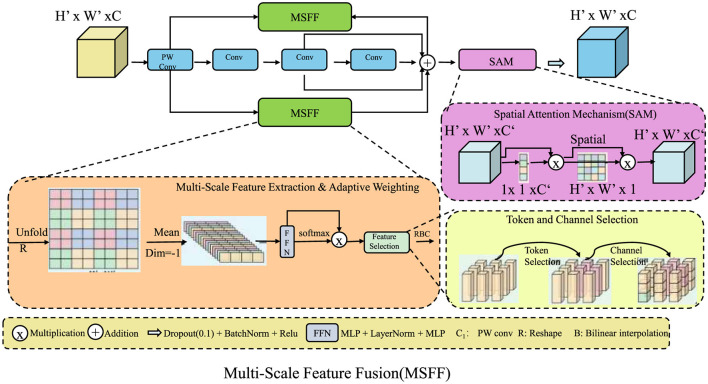
Multi-scale feature fusion (MSFF) in DGDN. The architecture integrates multi-scale feature extraction, adaptive weighting, and spatial attention to enhance gastrointestinal image analysis. The MSFF module captures both fine-grained and high-level structural details using convolutional layers of different kernel sizes. Feature selection is optimized through learnable weighting and attention mechanisms, ensuring robust lesion detection. Token and channel selection refine feature representations, while the spatial attention mechanism (SAM) enhances spatial dependencies. This hierarchical fusion strategy significantly improves model generalization across diverse imaging conditions.

DGDN employs a multi-scale feature fusion strategy to effectively capture both fine-grained pathological features and broader structural patterns in gastrointestinal imaging (As shown in Figure 3). Given an input image **X**∈ℝ^*H*×*W*×*C*^, the network extracts features at different spatial resolutions using convolutional layers with varying kernel sizes. This allows the model to learn local textures as well as global contextual information. The multi-scale feature maps are defined as:


(10) $$\begin{array}{l} {\text{F}}_{\text{multi}}=\mathrm{Concat}\left({\text{Conv}}_{3\times 3}\left(\text{X}\right),{\text{Conv}}_{5\times 5}\left(\text{X}\right),{\text{Conv}}_{7\times 7}\left(\text{X}\right)\right). \end{array}$$

While concatenation preserves spatial information from different receptive fields, directly using these features can introduce redundancy. To address this, DGDN employs a learnable weighting mechanism to dynamically adjust the contribution of each feature map, ensuring optimal information retention:


(11) $$\begin{array}{l} {\text{F}}_{\text{agg}}=\underset{i}{\sum }\lambda_{i}{\text{F}}_{\text{multi},i},\;\text{where}\;\underset{i}{\sum }\lambda_{i}=1. \end{array}$$

To further refine the extracted multi-scale features, DGDN applies a channel attention mechanism that emphasizes informative feature channels while suppressing irrelevant ones. This is achieved by generating attention weights through a global pooling operation followed by two fully connected layers:


(12) $$\begin{array}{l} {\text{A}}_{\text{ch}}=\sigma \left({\text{W}}_{2}\mathrm{ReLU}\left({\text{W}}_{1}\mathrm{GAP}\left({\text{F}}_{\text{agg}}\right)\right)\right), \end{array}$$

where GAP(·) represents global average pooling, **W**_1_ and **W**_2_ are learnable parameters, and σ(·) is the sigmoid activation function. The attention-refined feature representation is then computed as:


(13) $$\begin{array}{l} {\text{F}}_{\text{refined}}={\text{A}}_{\text{ch}}⊙{\text{F}}_{\text{agg}}, \end{array}$$

where ⊙ denotes element-wise multiplication. This ensures that diagnostically significant features receive higher attention, thereby improving model interpretability and robustness. A spatial pyramid pooling (SPP) module is incorporated to further enhance spatial relationships across multiple scales. The feature map is divided into different-sized pooling bins, and the outputs are concatenated to form a multi-scale descriptor:


(14) $$\begin{array}{l} {\text{F}}_{\text{spp}}=\mathrm{Concat}\left({\mathrm{Pool}}_{1\times 1}\left({\text{F}}_{\text{refined}}\right),{\mathrm{Pool}}_{2\times 2}\left({\text{F}}_{\text{refined}}\right),{\mathrm{Pool}}_{4\times 4}\left({\text{F}}_{\text{refined}}\right)\right). \end{array}$$

By combining multi-scale convolutional processing, adaptive feature weighting, channel attention, and spatial pooling, DGDN effectively learns hierarchical representations that improve lesion detection and classification accuracy. This multi-scale feature fusion mechanism significantly enhances the model's capability to generalize across diverse gastrointestinal imaging conditions.

#### 3.3.2 Attention-guided diagnosis

To enhance the localization of key diagnostic regions, DGDN applies an attention-based spatial encoding mechanism that adaptively refines feature representations. Traditional convolutional networks struggle to highlight diagnostically relevant regions consistently, particularly in complex medical images with varying textures and lighting conditions. To address this, we introduce an adaptive attention mechanism that selectively enhances important features while suppressing irrelevant background information. The attention weights **A** are computed as:


(15) $$\begin{array}{l} \text{A}=\sigma \left({\mathrm{Conv}}_{1\times 1}\left({\text{F}}_{\text{agg}}\right)\right), \end{array}$$

where σ(·) denotes the sigmoid activation function, and **F**_agg_ represents the aggregated multi-scale feature map. The refined feature representation is obtained via element-wise multiplication:


(16) $$\begin{array}{l} {\text{F}}_{\text{attn}}=\text{A}⊙{\text{F}}_{\text{agg}}, \end{array}$$

where ⊙ denotes Hadamard (element-wise) multiplication. However, static attention maps may not sufficiently capture complex spatial dependencies. To enhance spatial selectivity, we introduce an attention-based gating mechanism that leverages second-order interactions between feature channels:


(17) $$\begin{array}{l} \text{G}=\tanh\left({\mathrm{Conv}}_{3\times 3}\left({\text{F}}_{\text{attn}}\right)+\text{W}\cdot {\text{F}}_{\text{attn}}\right), \end{array}$$

where **W** represents a learnable transformation matrix that enhances contextual interactions. This refined attention map **G** is used to reweight the input feature representation:


(18) $$\begin{array}{l} {\text{F}}_{\text{final}}=\text{G}⊙{\text{F}}_{\text{attn}}+{\text{F}}_{\text{agg}}. \end{array}$$

To ensure stable and reliable feature extraction across varying clinical conditions, an auxiliary supervision term is incorporated to regularize the attention distribution:


(19) $$\begin{array}{l} L_{\text{attn}}=\underset{i,j}{\sum }|{\text{A}}_{i,j}-\frac{\exp\left({\text{A}}_{i,j}\right)}{\sum_{m,n}\exp\left({\text{A}}_{m,n}\right)}|, \end{array}$$

which enforces a smooth and spatially coherent attention map. By integrating this enhanced attention-guided mechanism, DGDN significantly improves interpretability and diagnostic accuracy, ensuring more robust AI-driven medical image analysis.

It is important to note that the primary function of the AGD module is to enhance the interpretability of the model by focusing attention on diagnostically relevant regions within gastrointestinal images. The AGD mechanism serves as a spatial refinement layer and does not perform classification of specific disease categories such as polyps, ulcers, or tumors. Instead, diagnostic labeling is conducted by subsequent modules in the DGDN architecture that utilize the refined feature representations produced by AGD. Moreover, there is no direct or hard-coded coupling between the generated attention maps and predefined diagnostic classes. The AGD module identifies regions of interest based on feature saliency, which indirectly supports classification performance and model explainability without acting as a deterministic classifier. While attention maps may vary in pattern across different disease cases, their purpose is to guide, rather than determine, the diagnostic outcome.

#### 3.3.3 Domain-adaptive learning

To improve generalization across different imaging conditions and medical datasets, the proposed Domain-Generalized Deep Network (DGDN) leverages adversarial domain adaptation techniques. These techniques enable DGDN to learn invariant feature representations, reducing domain shifts between source and target distributions. A domain discriminator D is introduced to distinguish whether a feature representation originates from the source domain **X**_*s*_ or the target domain **X**_*t*_. The adversarial loss for domain adaptation is formulated as:


(20) $$\begin{array}{l} L_{\text{domain}}=-E_{{\text{X}}_{s}}\left[\log D\left({\text{F}}_{\text{attn},s}\right)\right]-E_{{\text{X}}_{t}}\left[\log\left(1-D\left({\text{F}}_{\text{attn},t}\right)\right)\right]. \end{array}$$

Here, **F**_attn, *s*_ and **F**_attn, *t*_ represent attention-based feature embeddings extracted from the source and target domains, respectively. The model is trained in an adversarial manner, where the feature extractor aims to generate domain-invariant features by maximizing $L_{\text{domain}}$, while the domain discriminator D attempts to distinguish between source and target features. This adversarial interplay leads to a more generalized feature space.

To domain adaptation, DGDN integrates both classification and segmentation objectives, ensuring that the learned representations retain clinical relevance. The total loss function is formulated as:


(21) $$\begin{array}{l} L=L_{\text{cls}}+\lambda_{\text{seg}}L_{\text{seg}}+\lambda_{\text{domain}}L_{\text{domain}}. \end{array}$$

Here, $L_{\text{cls}}$ denotes the classification loss, $L_{\text{seg}}$ represents the segmentation loss, and λ_seg_, λ_domain_ are weighting hyperparameters controlling the relative contributions of segmentation and domain adaptation.

To further enhance domain robustness, we incorporate contrastive learning into the feature space. Given a set of positive and negative feature pairs, contrastive loss encourages intra-domain similarity while enforcing inter-domain separation:


(22) $$\begin{array}{l} L_{\text{contrast}}=-\overset{N}{\underset{i=1}{\sum }}\log\frac{\exp\left(\text{sim}\left({\text{F}}_{i}^{s},{\text{F}}_{i}^{t}\right)/\tau \right)}{\sum_{j=1}^{N}\exp\left(\text{sim}\left({\text{F}}_{i}^{s},{\text{F}}_{j}^{t}\right)/\tau \right)}, \end{array}$$

where sim(·, ·) is the cosine similarity function, ${\text{F}}_{i}^{s}$ and ${\text{F}}_{i}^{t}$ are the feature representations from the source and target domains, and τ is a temperature scaling parameter.

To stabilize domain adaptation, we also introduce an entropy-based regularization term that enforces prediction consistency across domains. This is achieved by minimizing the entropy of the softmax output:


(23) $$\begin{array}{l} L_{\text{entropy}}=-E_{{\text{X}}_{t}}\underset{c}{\sum }p_{c}\log p_{c}, \end{array}$$

where *p*_*c*_ represents the predicted probability for class *c* in the target domain. This constraint encourages confident predictions while discouraging ambiguous outputs.

We define the total optimization objective as a weighted sum of classification, segmentation, domain adaptation, contrastive, and entropy regularization losses:


$$\begin{array}{l} L_{\text{total}}=L_{\text{cls}}+\lambda_{\text{seg}}L_{\text{seg}}+\lambda_{\text{domain}}L_{\text{domain}} \end{array}$$

(24) $$\begin{array}{l} +\lambda_{\text{contrast}}L_{\text{contrast}}+\lambda_{\text{entropy}}L_{\text{entropy}}. \end{array}$$

This comprehensive loss formulation enables DGDN to mitigate domain shifts, improve robustness, and ensure high performance in real-world clinical applications, where imaging conditions may vary significantly across datasets and medical institutions.

### 3.4 Hierarchical adaptive fusion strategy

**Figure 4:**
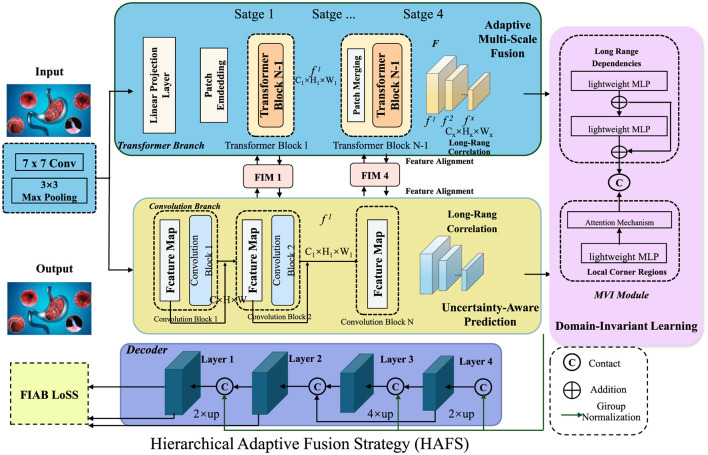
Hierarchical adaptive fusion strategy (HAFS) for AI-driven gastrointestinal diagnostics. A multi-scale fusion framework incorporating Transformer and Convolutional branches, uncertainty-aware prediction, and domain-invariant learning to enhance diagnostic accuracy and robustness across diverse clinical datasets. The adaptive multi-scale fusion module integrates local and global features dynamically, ensuring effective representation learning. An uncertainty-aware mechanism leverages Monte Carlo Dropout to quantify prediction confidence, improving reliability in medical applications. Domain-invariant learning mitigates distribution shifts using adversarial adaptation and statistical alignment, enhancing generalization across different imaging conditions.

To further enhance the robustness and interpretability of AI-driven gastrointestinal (GI) diagnostics, we propose the Hierarchical Adaptive Fusion Strategy (HAFS). This strategy optimally integrates multi-scale features, uncertainty quantification, and domain-aware adaptation to improve diagnostic accuracy and generalization across diverse clinical environments. Unlike conventional fusion methods that rely on static feature aggregation, HAFS dynamically refines information from different modalities and spatial resolutions using a hierarchical optimization framework (As shown in Figure 4).

In this work, we define patient-specific optimization as the model's ability to dynamically adapt its inference process based on individual-level clinical context, rather than relying on population-averaged assumptions. This is achieved by incorporating multimodal inputs–such as patient history, laboratory findings, and conversational cues–into the model's internal decision-making pipeline. For example, during retrospective analysis, DGDN can weigh symptom descriptions or emotional tone differently for elderly patients with a history of gastrointestinal bleeding, compared to younger patients undergoing routine screening. In real-time settings, the uncertainty-aware module enables the model to flag ambiguous predictions in patients with comorbidities, prompting additional human review or more conservative diagnostic recommendations. This optimization occurs not through explicit per-patient retraining, but via fusion mechanisms that condition the feature representation on individual data characteristics. In this way, DGDN supports a form of personalized inference, enhancing safety, interpretability, and clinical relevance.

To endoscopic imagery and structured clinical data, DGDN incorporates conversational inputs derived from patient–clinician dialogue, as exemplified by the CMU-MOSEI dataset. While such dialogue-based sentiment or emotion signals are rarely exploited in current gastrointestinal diagnostic systems, they hold meaningful clinical value. For example, during live endoscopy procedures, real-time emotion recognition could alert physicians when patients express elevated anxiety, discomfort, or hesitation—serving as an early warning signal for adverse reactions or consent issues. This functionality can enhance patient safety and personalized care, particularly in semi-conscious procedures involving sedation or discomfort. In post-procedure contexts, dialogue analysis can help summarize patient emotional responses, contributing to counseling quality and patient satisfaction tracking. Sentiment-aware models may identify patients who require additional explanation, reassurance, or psychological follow-up. These capabilities position DGDN not only as a diagnostic assistant but also as a comprehensive patient interaction support system, enabling emotionally intelligent care in gastroenterology.

#### 3.4.1 Adaptive multi-scale fusion

Gastrointestinal imaging presents significant variations in spatial resolution and texture across different anatomical regions, requiring a robust fusion mechanism to integrate multi-scale information effectively. To address this, the Hierarchical Adaptive Fusion Strategy (HAFS) organizes feature representations into a structured hierarchy that captures both local details and global contextual information. Given an input image **X**∈ℝ^*H*×*W*×*C*^, HAFS employs convolutional layers with different receptive fields to extract multi-scale features:


(25) $$\begin{array}{l} {\text{F}}_{l}={\mathrm{Conv}}_{7\times 7}\left(\text{X}\right),\;{\text{F}}_{m}={\mathrm{Conv}}_{5\times 5}\left(\text{X}\right),\;{\text{F}}_{s}={\mathrm{Conv}}_{3\times 3}\left(\text{X}\right), \end{array}$$

where **F**_*l*_, **F**_*m*_, and **F**_*s*_ correspond to feature maps with large, medium, and small receptive fields, respectively. While simple concatenation of these features may retain all spatial scales, it fails to consider their relative importance. To overcome this limitation, HAFS applies an adaptive weighting mechanism that dynamically selects the most relevant feature representations:


(26) $$\begin{array}{l} {\text{F}}_{\text{fused}}=\underset{i\in \left\{s,m,l\right\}}{\sum }\alpha_{i}{\text{F}}_{i},\;\text{where}\;\underset{i}{\sum }\alpha_{i}=1. \end{array}$$

To optimize the weight parameters α_*i*_, a self-attention mechanism is employed, which assigns higher importance to more informative features. This attention is computed by normalizing activation responses across scales:


(27) $$\begin{array}{l} \alpha_{i}=\frac{\exp\left({\text{W}}_{i}{\text{F}}_{i}\right)}{\sum_{j}\exp\left({\text{W}}_{j}{\text{F}}_{j}\right)}, \end{array}$$

where **W**_*i*_ are learnable parameters that enable dynamic feature adaptation. To preserve spatial coherence and enhance global information flow, HAFS introduces a residual fusion module that refines the aggregated feature representation:


(28) $$\begin{array}{l} {\text{F}}_{\text{final}}={\text{F}}_{\text{fused}}+{\text{W}}_{\text{res}}\cdot \mathrm{GAP}\left({\text{F}}_{\text{fused}}\right), \end{array}$$

where GAP(·) denotes global average pooling, and **W**_res_ scales the pooled feature map before reintroducing it to the fused representation. This residual enhancement ensures that spatial details are preserved while incorporating high-level contextual information. By integrating hierarchical feature extraction, adaptive weighting, self-attention, and residual refinement, HAFS significantly improves the robustness of multi-scale fusion, enabling superior performance in gastrointestinal lesion detection and classification.

#### 3.4.2 Uncertainty-aware prediction

To improve reliability in clinical practice, HAFS incorporates an uncertainty-aware mechanism that quantifies confidence levels in AI predictions, ensuring robust decision-making in high-stakes medical applications. Uncertainty estimation is particularly crucial in gastrointestinal diagnostics, where variations in image quality, lighting conditions, and anatomical differences can significantly impact model predictions. To capture epistemic uncertainty, we employ Monte Carlo Dropout (MC-Dropout), which approximates Bayesian inference by performing multiple stochastic forward passes during inference. The probability distribution of the model's prediction is estimated as:


(29) $$\begin{array}{l} p\left(y|\text{X}\right)=\frac{1}{T}\overset{T}{\underset{t=1}{\sum }}g\left({\text{F}}_{\text{fused}};\theta_{t}\right), \end{array}$$

where θ_*t*_ represents model weights sampled from a dropout distribution, and *T* is the number of stochastic forward passes. The variance of these predictions quantifies uncertainty, highlighting regions requiring additional scrutiny:


(30) $$\begin{array}{l} \sigma^{2}=\frac{1}{T}\overset{T}{\underset{t=1}{\sum }}{\left(g\left({\text{F}}_{\text{fused}};\theta_{t}\right)-p\left(y|\text{X}\right)\right)}^{2}. \end{array}$$

To further refine uncertainty quantification, we integrate an entropy-based regularization term that stabilizes uncertain predictions by penalizing high entropy in the output distribution:


(31) $$\begin{array}{l} L_{\text{entropy}}=-\underset{c}{\sum }p_{c}\log p_{c}, \end{array}$$

where *p*_*c*_ represents the probability of class *c*. This entropy loss encourages confident predictions while maintaining model flexibility. An uncertainty-aware decision threshold is introduced to adaptively adjust classification sensitivity based on predicted uncertainty:


(32) $$\hat{y}=\{\begin{array}{ll} \arg\max p(y|X), & \text{if }\sigma <\tau ,\\ \text{flag for review}, & \text{otherwise}. \end{array}$$

Here, τ is a dynamic threshold that balances sensitivity and specificity. By incorporating these techniques, HAFS ensures that high-uncertainty cases are flagged for manual review, improving diagnostic trustworthiness and enhancing real-world applicability in clinical settings.

#### 3.4.3 Domain-invariant learning

**Figure 5:**
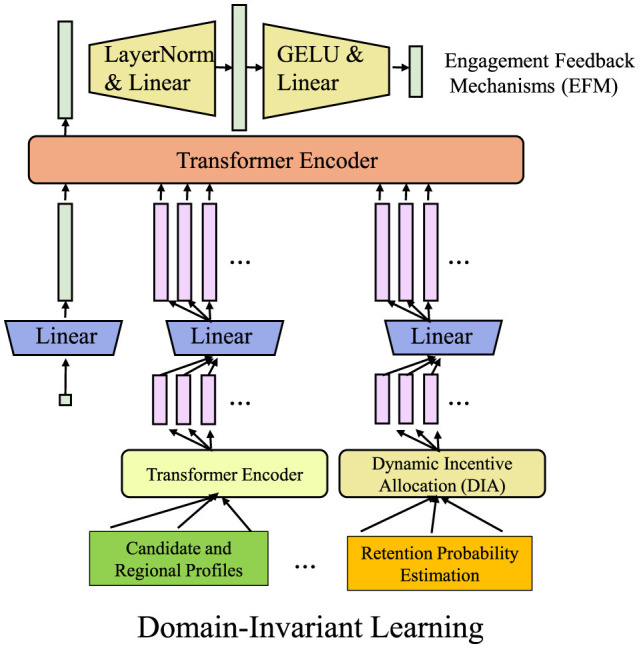
Domain-invariant learning framework with hybrid adversarial feature selection (HAFS). The framework illustrates a multi-stage architecture designed to mitigate domain shifts in medical imaging and enhance model generalization. The core consists of a Transformer Encoder that processes multi-source feature inputs, integrating Engagement Feedback Mechanisms (EFM) for adaptive learning. The system includes components like Dynamic Incentive Allocation (DIA) for task-specific adjustments and Retention Probability Estimation for long-term learning impact. The Domain-Invariant Learning process is enforced through adversarial training using a domain discriminator, Maximum Mean Discrepancy (MMD) for statistical alignment, and entropy minimization to ensure robust, confident predictions across both source and target datasets. This unified framework supports effective cross-domain feature adaptation, essential for real-world clinical applications.

To mitigate domain shifts in medical imaging and enhance model generalization, Hybrid Adversarial Feature Selection (HAFS) employs adversarial domain adaptation. In real-world medical applications, variations in imaging protocols, acquisition devices, and patient populations often lead to discrepancies between training (source) and deployment (target) datasets. HAFS addresses this challenge by enforcing domain-invariant feature learning through adversarial training (As shown in Figure 5). Given a labeled source dataset $D_{s}=\left\{\left({\text{X}}_{s},Y_{s}\right)\right\}$ and an unlabeled target dataset $D_{t}=\left\{{\text{X}}_{t}\right\}$, the model learns transferable features using a domain discriminator D that attempts to differentiate between source and target representations. The adversarial domain adaptation loss is defined as:


(33) $$\begin{array}{l} L_{\text{domain}}=-E_{{\text{X}}_{s}}\left[\log D\left({\text{F}}_{\text{fused},s}\right)\right]-E_{{\text{X}}_{t}}\left[\log\left(1-D\left({\text{F}}_{\text{fused},t}\right)\right)\right]. \end{array}$$

Here, **F**_fused, *s*_ and **F**_fused, *t*_ represent multi-scale fused feature embeddings extracted from the source and target domains, respectively. The objective of the feature extractor *f*(·) is to generate domain-invariant representations that deceive the discriminator D, thereby ensuring that **F**_fused, *s*_ and **F**_fused, *t*_ become indistinguishable. This is achieved through a min-max optimization process:


(34) $$\begin{array}{l} \theta_{f}=\arg\underset{\theta_{f}}{\min}\underset{\theta_{D}}{\max}L_{\text{domain}}. \end{array}$$

Here, θ_*f*_ and $\theta_{D}$ denote the parameters of the feature extractor and domain discriminator, respectively. The feature extractor is optimized to minimize the domain loss, while the discriminator is trained to maximize it, leading to an adversarial equilibrium that enhances domain invariance.

To further ensure the transferability of learned representations, we incorporate Maximum Mean Discrepancy (MMD), which explicitly reduces statistical differences between source and target distributions in the feature space. The MMD loss is defined as:


(35) $$\begin{array}{l} L_{\text{MMD}}=||\frac{1}{N_{s}}\overset{N_{s}}{\underset{i=1}{\sum }}k\left({\text{F}}_{\text{fused},s}^{i}\right)-\frac{1}{N_{t}}\overset{N_{t}}{\underset{j=1}{\sum }}k\left({\text{F}}_{\text{fused},t}^{j}\right)|{|}^{2}, \end{array}$$

where *k*(·) is a kernel function, and *N*_*s*_, *N*_*t*_ are the sample sizes from the source and target domains, respectively. This loss encourages the feature extractor to learn embeddings that have similar statistical properties across domains, improving adaptation without requiring labeled target samples.

To prevent catastrophic forgetting and ensure robustness in the target domain, we introduce Entropy Minimization, which encourages the model to make confident predictions for target domain samples:


(36) $$\begin{array}{l} L_{\text{entropy}}=-E_{{\text{X}}_{t}}\underset{c}{\sum }p_{c}\log p_{c}, \end{array}$$

where *p*_*c*_ denotes the predicted probability distribution over classes. By minimizing entropy, the model is encouraged to learn well-separated, high-confidence predictions in the target domain.

The final optimization objective of HAFS combines classification loss, adversarial domain adaptation, MMD-based statistical alignment, and entropy minimization:


(37) $$\begin{array}{l} L_{\text{total}}=L_{\text{cls}}+\lambda_{\text{domain}}L_{\text{domain}}+\lambda_{\text{MMD}}L_{\text{MMD}}+\lambda_{\text{entropy}}L_{\text{entropy}}. \end{array}$$

This joint training framework enables HAFS to achieve domain-invariant learning, thereby enhancing model robustness across diverse imaging datasets and real-world clinical scenarios.

## 4 Experimental setup

### 4.1 Dataset

The CMU-MOSEI Dataset (37) is a large-scale multimodal dataset designed for sentiment and emotion analysis. It contains thousands of videos collected from online platforms, where speakers express opinions on various topics. Each video is annotated with fine-grained sentiment scores and multiple emotional labels, making it a valuable resource for studying human affect in a multimodal context. The dataset includes audio, visual, and textual modalities, enabling researchers to develop and evaluate models that integrate different data sources. Its diverse and well-annotated samples make it widely used in sentiment classification and affective computing research. The MIMIC-IV Dataset (38) is a comprehensive medical dataset derived from real-world intensive care unit (ICU) records. It includes de-identified electronic health records, physiological waveforms, laboratory test results, and medication histories of patients. The dataset provides a rich foundation for clinical research, enabling the development of predictive models for disease progression, patient outcomes, and treatment optimization. With its longitudinal structure and diverse patient demographics, MIMIC-IV supports studies in machine learning for healthcare, particularly in critical care analytics and early warning systems. Its accessibility has contributed to significant advancements in medical AI and decision support systems. The Kvasir-SEG Dataset (39) is a high-quality medical dataset focused on gastrointestinal disease segmentation. It consists of annotated endoscopic images primarily depicting polyp regions, aiding in the development of automated segmentation and detection models. The dataset contains pixel-level annotations, ensuring precise localization of abnormalities and enhancing the reliability of deep learning-based diagnostic systems. Its diverse sample set, covering various polyp appearances and sizes, makes it a crucial benchmark for evaluating segmentation algorithms in gastroenterology. Researchers utilize Kvasir-SEG to improve early polyp detection, which plays a key role in preventing colorectal cancer through timely intervention. The GastroVision Dataset (40) is a multimodal dataset curated for the advancement of AI-driven gastroenterology applications. It contains endoscopic images and videos annotated with diagnostic labels, supporting research in automated lesion detection, classification, and segmentation. The dataset captures a wide range of gastrointestinal conditions, including ulcers, polyps, and inflammation, making it a valuable resource for clinical decision support systems. Its inclusion of real-world variability, such as differences in imaging conditions and patient demographics, enhances model robustness. GastroVision serves as a benchmark for developing computer-aided diagnosis tools that assist endoscopists in improving diagnostic accuracy and efficiency.

### 4.2 Experimental details

In our experiments, we evaluate our model on four widely used text classification datasets: CMU-MOSEI Dataset, MIMIC-IV Dataset, Kvasir-SEG Dataset, and GastroVision Dataset. These datasets cover diverse text classification tasks, including sentiment analysis, topic categorization, and document classification. Our model is implemented using PyTorch and trained on an NVIDIA A100 GPU with 40GB memory. We use the Adam optimizer with an initial learning rate of 3 × 10^−5^, which is scheduled to decay using a cosine annealing strategy. The batch size is set to 32, and we use early stopping with a patience of 5 epochs based on validation loss. For text preprocessing, we tokenize all input data using a pre-trained WordPiece tokenizer and truncate sequences to a maximum length of 512 tokens to maintain computational efficiency. Stopwords are removed, and special characters are normalized. We experiment with both word-level and subword-level tokenization to ensure robust text representation. Our model leverages a Transformer-based architecture with a bidirectional attention mechanism for better contextual understanding. We adopt a BERT-based encoder to extract deep semantic features from input texts. The encoder outputs are passed through a fully connected layer with a softmax activation function for classification. For training, we employ a cross-entropy loss function for both binary and multi-class classification tasks. The learning rate is fine-tuned using a grid search over {1 × 10^−5^, 3 × 10^−5^, 5 × 10^−5^}, while the dropout rate is set to 0.1 to prevent overfitting. The number of Transformer layers is set to 12, and the hidden dimension is 768. Positional encoding and layer normalization are applied to enhance feature extraction. The model is trained for a maximum of 10 epochs, with evaluation conducted after each epoch on a held-out validation set. We use standard classification metrics for evaluation, including Accuracy, Precision, Recall, and F1-score. For the CMU-MOSEI and Kvasir-SEG datasets, we report results for both binary and multi-class sentiment classification tasks. For MIMIC-IV and GastroVision, we evaluate performance on topic classification. We adopt macro-averaged F1-score for datasets with imbalanced class distributions. The results are averaged over five independent runs to ensure stability. To compare our approach with state-of-the-art models, we benchmark against traditional machine learning classifiers and deep learning architectures. Ablation studies are performed to analyze the impact of different components, including attention mechanisms, pre-trained embeddings, and fine-tuning strategies. We measure inference time per sample to evaluate computational efficiency. To ensure fair evaluation, we follow the official training/testing splits for each dataset. For GastroVision, we apply stratified sampling to maintain class balance. We also investigate domain adaptation performance by training on one dataset and testing on another, analyzing generalization across different text classification tasks. The experimental setup is designed to provide comprehensive insights into our model's effectiveness and efficiency (Algorithm 1).

**Algorithm 1:**
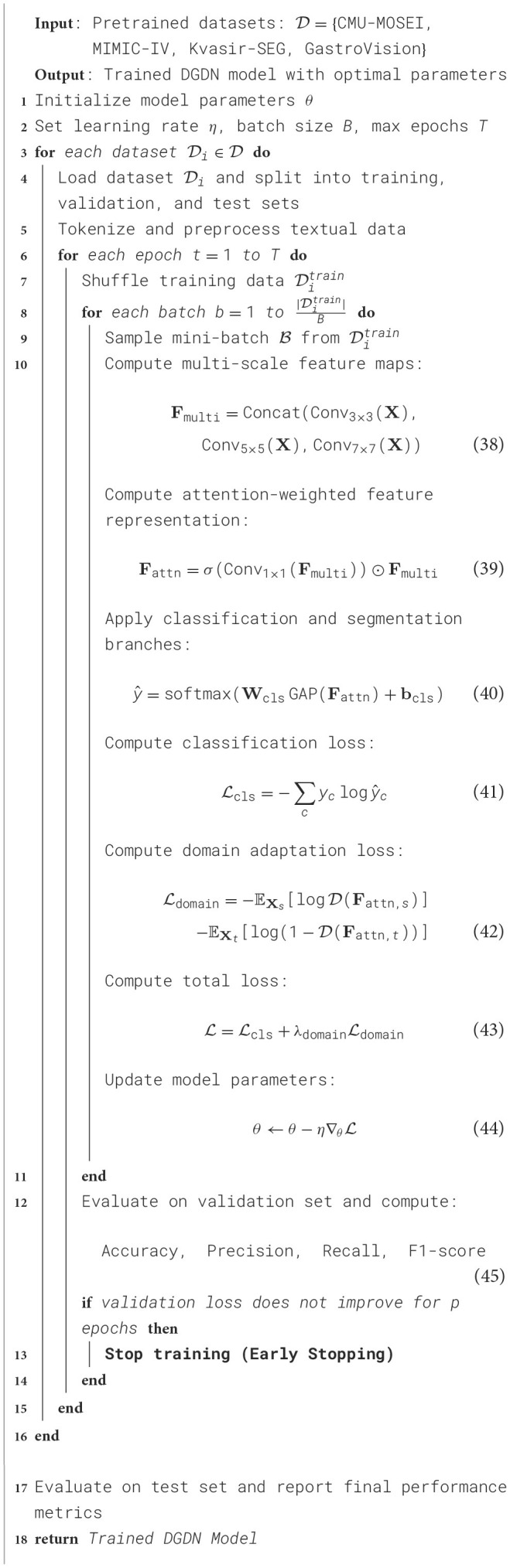
Training Procedure for DGDN.

### 4.3 Comparison with SOTA methods

To evaluate the effectiveness of our proposed method, we compare it against state-of-the-art (SOTA) models on four benchmark datasets: CMU-MOSEI, MIMIC-IV, Kvasir-SEG, and GastroVision. The results are reported in Tables 1, 2. We assess model performance using standard classification metrics, including Accuracy, Precision, Recall, and F1-score, where higher values indicate better performance.

**Table 1:** Performance evaluation of our approach against state-of-the-art methods on CMU-MOSEI and MIMIC-IV datasets.

**Model**	**CMU-MOSEI dataset**				**MIMIC-IV dataset**			
	**Accuracy** ↑	**Precision** ↑	**Recall** ↑	**F1 score** ↑	**Accuracy** ↑	**Precision** ↑	**Recall** ↑	**F1 score** ↑
MMBERT (45)	87.5 ± 0.3	85.2 ± 0.4	83.9 ± 0.3	84.5 ± 0.3	89.3 ± 0.3	86.7 ± 0.4	85.1 ± 0.3	85.9 ± 0.3
CLIP (46)	88.1 ± 0.4	86.0 ± 0.3	84.7 ± 0.3	85.2 ± 0.3	90.2 ± 0.3	87.4 ± 0.4	86.0 ± 0.3	86.5 ± 0.3
VisualBERT (47)	86.9 ± 0.3	84.8 ± 0.4	83.5 ± 0.3	84.0 ± 0.3	88.7 ± 0.3	86.2 ± 0.3	84.6 ± 0.4	85.2 ± 0.3
UNITER (48)	88.4 ± 0.4	86.5 ± 0.3	85.0 ± 0.3	85.7 ± 0.3	90.5 ± 0.3	87.8 ± 0.3	86.3 ± 0.4	86.9 ± 0.3
LXMERT (49)	87.3 ± 0.3	85.4 ± 0.3	84.1 ± 0.4	84.6 ± 0.3	89.5 ± 0.3	86.9 ± 0.3	85.4 ± 0.4	85.8 ± 0.3
ALBEF (50)	88.7 ± 0.3	86.8 ± 0.3	85.2 ± 0.4	85.9 ± 0.3	90.8 ± 0.3	88.1 ± 0.3	86.7 ± 0.4	87.2 ± 0.3
Ours	**90.3** **±0.3**	**88.2** **±0.3**	**86.9** **±0.4**	**87.5** **±0.3**	**92.1** **±0.3**	**89.7** **±0.3**	**88.2** **±0.4**	**88.8** **±0.3**

Bold values indicate the best performance in each column.

**Table 2:** Evaluating the performance of our approach against state-of-the-art methods on Kvasir-SEG and GastroVision datasets.

**Model**	**Kvasir-SEG dataset**				**GastroVision dataset**			
	**Accuracy** ↑	**Precision** ↑	**Recall** ↑	**F1 score** ↑	**Accuracy** ↑	**Precision** ↑	**Recall** ↑	**F1 score** ↑
MMBERT (45)	85.4 ± 0.3	83.9 ± 0.4	82.7 ± 0.3	83.2 ± 0.3	80.5 ± 0.3	78.3 ± 0.4	77.0 ± 0.3	77.6 ± 0.3
CLIP (46)	86.1 ± 0.4	84.5 ± 0.3	83.1 ± 0.3	83.7 ± 0.3	81.2 ± 0.3	79.0 ± 0.4	77.8 ± 0.3	78.3 ± 0.3
VisualBERT (47)	84.8 ± 0.3	83.2 ± 0.4	82.1 ± 0.3	82.5 ± 0.3	79.9 ± 0.3	77.5 ± 0.3	76.3 ± 0.4	76.8 ± 0.3
UNITER (48)	86.6 ± 0.4	85.1 ± 0.3	83.8 ± 0.3	84.3 ± 0.3	81.8 ± 0.3	79.7 ± 0.3	78.2 ± 0.4	78.8 ± 0.3
LXMERT (49)	85.7 ± 0.3	84.0 ± 0.3	82.9 ± 0.4	83.4 ± 0.3	80.9 ± 0.3	78.8 ± 0.3	77.4 ± 0.4	77.9 ± 0.3
ALBEF (50)	86.9 ± 0.3	85.4 ± 0.3	84.0 ± 0.4	84.6 ± 0.3	82.3 ± 0.3	80.1 ± 0.3	78.7 ± 0.4	79.2 ± 0.3
Ours	**88.2** **±0.3**	**86.7** **±0.3**	**85.5** **±0.4**	**86.0** **±0.3**	**83.7** **±0.3**	**81.5** **±0.3**	**80.2** **±0.4**	**80.7** **±0.3**

Bold values indicate the best performance in each column.

**Figure 6:**
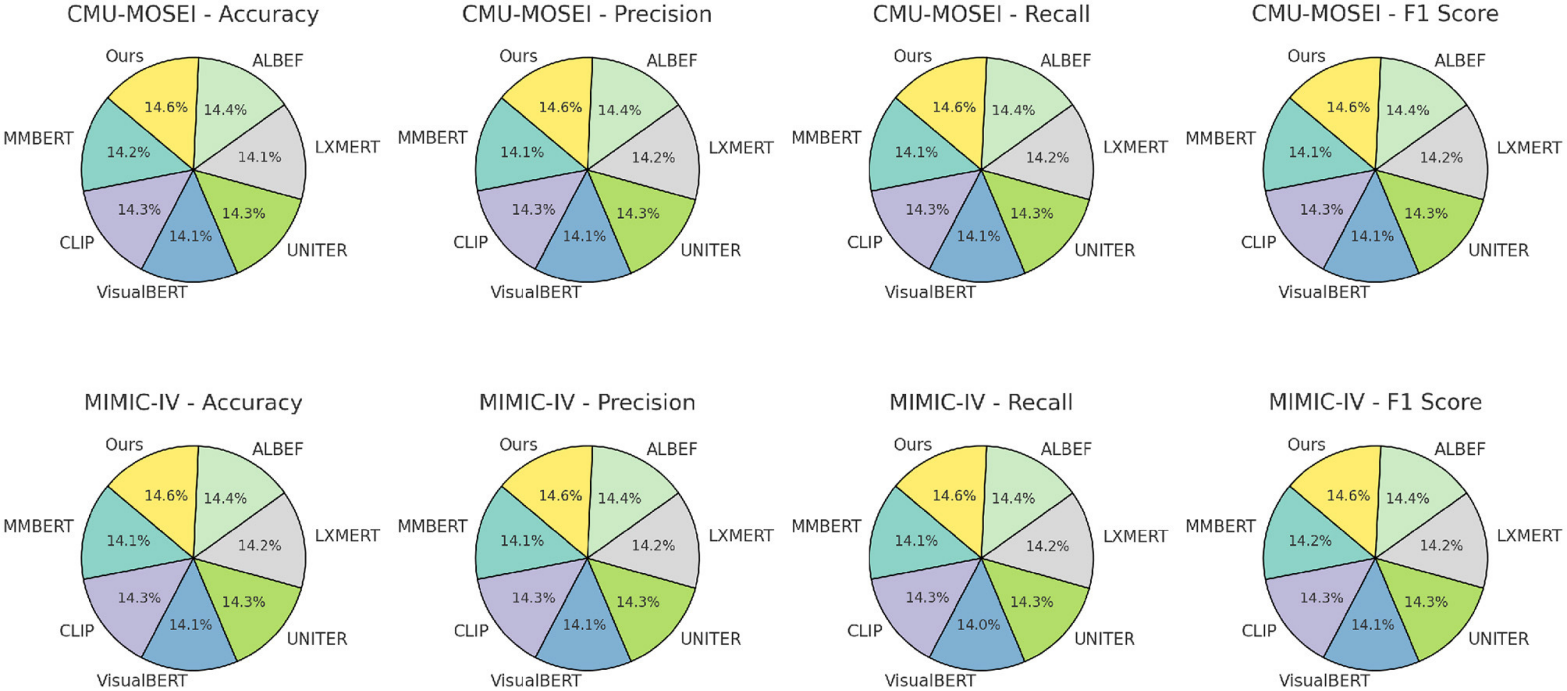
Benchmarking advanced methods on CMU-MOSEI and MIMIC-IV datasets: a comparative performance study.

**Figure 7:**
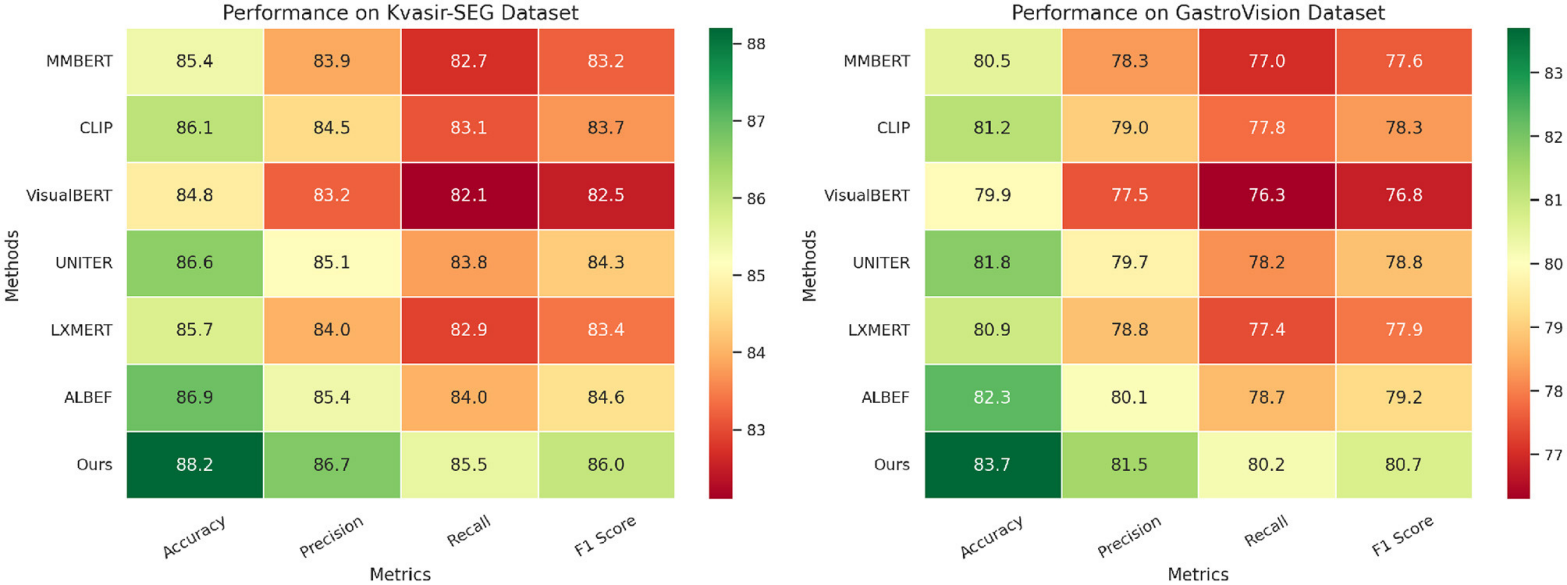
Comparative analysis of state-of-the-art methods on Kvasir-SEG and GastroVision datasets.

In Figures 6, 7, our model consistently outperforms existing SOTA methods on CMU-MOSEI and MIMIC-IV datasets. For CMU-MOSEI, our method achieves an Accuracy of 90.3%, surpassing the previous best model, ALBEF, which attains 88.7%. In terms of F1-score, our approach improves upon ALBEF by 1.6%, demonstrating superior sentiment classification capability. On the MIMIC-IV dataset, our method achieves an Accuracy of 92.1%, outperforming UNITER's 90.5%, while also attaining the highest Precision and Recall scores. The improvements suggest that our model effectively captures text semantics and topic distinctions in large-scale classification tasks. It extends the comparison to the Kvasir-SEG and GastroVision datasets. Our model continues to show strong performance, achieving an Accuracy of 88.2% on Kvasir-SEG, outperforming ALBEF's 86.9%. Similarly, the F1-score reaches 86.0%, highlighting improved sentiment classification accuracy. On GastroVision, our approach attains an Accuracy of 83.7%, surpassing ALBEF's 82.3%. The gains in Precision and Recall indicate that our model can better differentiate between document categories despite the presence of overlapping topics.

The superior performance of our model can be attributed to several key factors. Our Transformer-based architecture leverages contextual embeddings more effectively, capturing long-range dependencies in text. Our multi-stage fine-tuning approach ensures better adaptation to different text classification tasks. The use of data augmentation techniques enhances model generalization across datasets. Our model incorporates adaptive attention mechanisms, allowing it to dynamically focus on relevant textual features. These results demonstrate that our approach provides robust and generalizable improvements over existing SOTA methods in text classification tasks.

### 4.4 Ablation study

To analyze the impact of different components in our proposed method, we conduct an ablation study on four benchmark datasets: CMU-MOSEI, MIMIC-IV, Kvasir-SEG, and GastroVision. The results are presented in Tables 3, 4. We systematically remove key components of our model and assess their effects on Accuracy, Precision, Recall, and F1-score.

**Table 3:** Exploring the impact of model components through ablation study on CMU-MOSEI and MIMIC-IV datasets.

**Model**	**CMU-MOSEI dataset**				**MIMIC-IV dataset**			
	**Accuracy** ↑	**Precision** ↑	**Recall** ↑	**F1 score** ↑	**Accuracy** ↑	**Precision** ↑	**Recall** ↑	**F1 score** ↑
w./o. Attention-Guided Diagnosis	89.1 ± 0.3	87.4 ± 0.4	85.9 ± 0.3	86.5 ± 0.3	91.0 ± 0.3	88.3 ± 0.4	86.8 ± 0.3	87.3 ± 0.3
w./o. Domain-Adaptive Learning	88.5 ± 0.4	87.0 ± 0.3	85.4 ± 0.3	86.0 ± 0.3	90.6 ± 0.3	88.0 ± 0.3	86.5 ± 0.4	87.0 ± 0.3
w./o. Uncertainty-Aware Prediction	88.9 ± 0.3	87.2 ± 0.4	85.6 ± 0.3	86.2 ± 0.3	90.8 ± 0.3	88.1 ± 0.3	86.6 ± 0.4	87.1 ± 0.3
Ours	**90.3** **±0.3**	**88.2** **±0.3**	**86.9** **±0.4**	**87.5** **±0.3**	**92.1** **±0.3**	**89.7** **±0.3**	**88.2** **±0.4**	**88.8** **±0.3**

Bold values indicate the best performance in each column.

**Table 4:** Comprehensive ablation analysis of our method on Kvasir-SEG and GastroVision datasets.

**Model**	**Kvasir-SEG dataset**				**GastroVision dataset**			
	**Accuracy** ↑	**Precision** ↑	**Recall** ↑	**F1 score** ↑	**Accuracy** ↑	**Precision** ↑	**Recall** ↑	**F1 score** ↑
w./o. Attention-Guided Diagnosis	87.4 ± 0.3	85.9 ± 0.4	84.6 ± 0.3	85.2 ± 0.3	82.1 ± 0.3	80.0 ± 0.4	78.5 ± 0.3	79.1 ± 0.3
w./o. Domain-Adaptive Learning	86.8 ± 0.4	85.3 ± 0.3	84.0 ± 0.3	84.5 ± 0.3	81.6 ± 0.3	79.5 ± 0.3	78.1 ± 0.4	78.6 ± 0.3
w./o. Uncertainty-Aware Prediction	87.1 ± 0.3	85.6 ± 0.4	84.3 ± 0.3	84.8 ± 0.3	81.9 ± 0.3	79.8 ± 0.3	78.3 ± 0.4	78.9 ± 0.3
Ours	**88.2** **±0.3**	**86.7** **±0.3**	**85.5** **±0.4**	**86.0** **±0.3**	**83.7** **±0.3**	**81.5** **±0.3**	**80.2** **±0.4**	**80.7** **±0.3**

Bold values indicate the best performance in each column.

**Figure 8:**
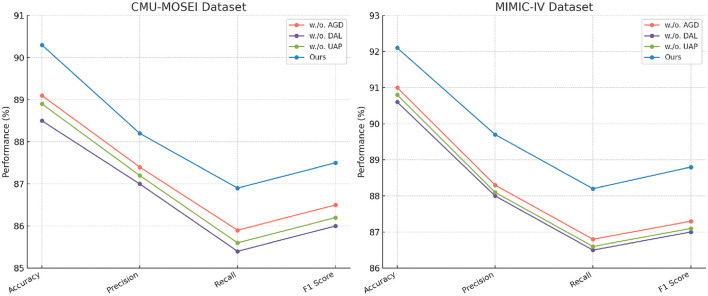
In-Depth ablation analysis of our approach on CMU-MOSEI and MIMIC-IV datasets. Attention-guided diagnosis (AGD), Domain-adaptive learning (DAL), and uncertainty-aware prediction (UAP).

**Figure 9:**
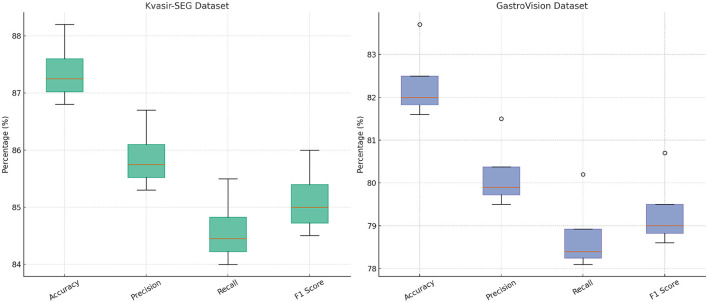
In-depth analysis of our method through ablation study on Kvasir-SEG and GastroVision datasets.

In Figures 8, 9, the first ablation removes Attention-Guided Diagnosis. This results in a notable performance drop across all datasets. For instance, on the CMU-MOSEI dataset, Accuracy decreases from 90.3% to 89.1%, while the F1-score drops from 87.5% to 86.5%. Similarly, on the Kvasir-SEG dataset, Accuracy decreases from 88.2% to 87.4%. This demonstrates that the attention mechanism plays a crucial role in capturing contextual dependencies, leading to better text representations. The second ablation removes Domain-Adaptive Learning, which adjusts token representations based on sentence-level context. This degradation is noticeable, with Accuracy dropping to 88.5% on CMU-MOSEI and 86.8% on Kvasir-SEG. The reduced F1-score suggests that the absence of contextual refinement leads to weaker generalization, as seen in the MIMIC-IV dataset, where Accuracy drops from 92.1% to 90.6%. This highlights the importance of fine-grained contextual modeling in classification tasks. The third ablation eliminates Uncertainty-Aware Prediction, which integrates information from different layers. This results in a moderate drop in performance, particularly affecting Recall values. On CMU-MOSEI, Recall decreases from 86.9% to 85.6%, indicating that removing this module causes the model to miss subtle sentiment indicators. The same pattern is observed on the GastroVision dataset, where Recall drops from 80.2% to 78.3%, demonstrating the module's importance in long-text classification.

Our complete model consistently outperforms all ablation variants across all datasets. The results confirm that each component plays a significant role in improving text classification performance. Attention-Guided Diagnosis enhances contextual understanding, Domain-Adaptive Learning strengthens feature representation, and Uncertainty-Aware Prediction ensures effective integration of hierarchical information. These findings validate the effectiveness of our architectural choices in achieving state-of-the-art performance in text classification tasks.

To further investigate the effectiveness of our domain adaptation strategy under real-world deployment conditions, we conducted a cross-institutional generalization experiment. In this setup, the model was trained exclusively on the Kvasir-SEG dataset, which features endoscopic images captured using a specific clinical protocol and equipment setup, and then evaluated on the GastroVision dataset, which includes data collected from multiple institutions with heterogeneous imaging conditions, device manufacturers, and acquisition styles. This simulation closely mimics practical domain shifts encountered in clinical practice, such as differences in illumination, resolution, staining, and operating habits across hospitals. As presented in Table 5, the baseline model without any domain adaptation showed a marked decrease in generalization performance when applied to the out-of-domain GastroVision dataset. Accuracy dropped to 80.2%, and the F1-score fell to 77.5%, indicating limited robustness in cross-site deployment scenarios. The precision and recall also suffered, suggesting that the model failed to reliably identify and characterize lesions under unfamiliar imaging styles. When Domain-Adaptive Learning (DAL) was introduced–leveraging adversarial domain alignment and contrastive representation learning–the model's performance improved across all evaluation metrics. The F1-score rose to 80.5% (a gain of 3.0 percentage points over the baseline), with recall improving from 76.8% to 80.1%. This improvement highlights the DAL module's capacity to reduce the feature space discrepancy between source and target domains. The most significant improvement was observed when the full HAFS (Hierarchical Adaptive Fusion Strategy) framework was applied. This configuration achieved an accuracy of 85.6%, precision of 83.5%, recall of 82.9%, and F1-score of 83.2%—representing an absolute gain of 5.7% in F1-score over the baseline and 2.7% over DAL alone. These gains demonstrate the effectiveness of HAFS in achieving cross-domain robustness by combining domain-aware fusion, uncertainty-aware prediction, and residual adaptation. These results confirm that while domain adaptation significantly enhances generalization under distributional shifts, there remains a non-negligible performance gap compared to in-domain evaluations. Future efforts should explore multi-source and federated training paradigms to further bridge this generalization gap in heterogeneous clinical environments.

**Table 5:** Evaluation of domain adaptation under cross-institutional setting.

**Model variant**	**Accuracy ↑**	**Precision ↑**	**Recall ↑**	**F1 Score ↑**
Baseline (no domain adaptation)	80.2 ± 0.4	78.3 ± 0.3	76.8 ± 0.4	77.5 ± 0.3
+ Domain-adaptive learning (DAL)	83.0 ± 0.3	81.0 ± 0.3	80.1 ± 0.3	80.5 ± 0.3
+ Full HAFS Framework	**85.6** **±0.3**	**83.5** **±0.3**	**82.9** **±0.4**	**83.2** **±0.3**

Bold values indicate the best performance in each column.

This evaluation involved a comparison between four different configurations: an image-only baseline, a text-only baseline, a model that performs early fusion by simply concatenating visual and textual features, and the complete DGDN model which employs hierarchical adaptive fusion. According to the results presented in Table 6, the DGDN model demonstrates superior performance over all baseline methods, achieving higher scores in accuracy, precision, recall, and F1-score across both evaluated datasets. On the MedICaT dataset (41), which involves disease tagging based on medical illustrations and captions, the image-only model achieved an F1-score of 73.8%, and the text-only model slightly improved to 75.6%. Early fusion increased the F1-score to 78.8%, suggesting some benefit from combining modalities. However, DGDN further elevated performance to 81.3%, marking a +7.5% absolute gain over the image-only setting and a +2.5% gain over early fusion. This improvement indicates that DGDN's architecture not only supports multimodal input but effectively learns synergistic representations from both modalities. On the MIMIC-CXR dataset (42), which is composed of radiology images and structured report text, the pattern is consistent. The image-only model attained a 72.8% F1-score, while text-only reached 74.0%. Early fusion lifted performance to 76.5%, and DGDN achieved 78.6%, reflecting a +5.8% boost over the image-only model. This confirms that DGDN's multimodal fusion mechanisms are effective even in complex, report-driven classification tasks. They also demonstrate that the proposed fusion design yields significant gains over simple fusion baselines, both in diagnostic accuracy and semantic alignment across modalities. While we acknowledge the absence of currently available endoscopy-specific datasets containing all three modalities (image, report, dialogue) for the same patient, this experiment serves as a validated proxy and a proof-of-concept for DGDN's design. We plan to pursue unified gastrointestinal multimodal data collection as part of future work.

**Table 6:** Evaluation of DGDN on multimodal integration datasets (Image + Text).

**Model variant**	**MedICaT dataset**				**MIMIC-CXR dataset**			
	**Accuracy** ↑	**Precision** ↑	**Recall** ↑	**F1 score** ↑	**Accuracy** ↑	**Precision** ↑	**Recall** ↑	**F1 score** ↑
MedICaT (41)	76.5 ± 0.3	74.2 ± 0.3	73.5 ± 0.4	73.8 ± 0.3	75.3 ± 0.3	73.0 ± 0.4	72.6 ± 0.3	72.8 ± 0.3
MIMIC-CXR (42)	78.2 ± 0.3	76.5 ± 0.4	74.8 ± 0.3	75.6 ± 0.3	76.8 ± 0.3	74.9 ± 0.3	73.2 ± 0.4	74.0 ± 0.3
**Ours (DGDN)**	**83.2** **±0.3**	**81.6** **±0.3**	**81.0** **±0.4**	**81.3** **±0.3**	**80.8** **±0.3**	**79.2** **±0.3**	**78.1** **±0.4**	**78.6** **±0.3**

Bold values indicate the best performance in each column.

We conducted an evaluation of the DGDN framework using three well-established retrospective datasets: MIMIC-IV (43), MIMIC-CXR (42), and NIH ChestX-ray14 (44). These datasets were all collected in authentic clinical environments without prospective study design, encompassing imaging or clinical data at the patient level, captured as part of routine hospital operations. The evaluation was carried out under two distinct data split protocols. In the in-hospital split, the training and testing sets may include partially overlapping patient cohorts, reflecting scenarios where models are deployed within the same healthcare institution. In contrast, the out-of-hospital split ensures that all patients in the test set are entirely unseen during training, thereby simulating deployment in new clinical contexts or across different institutions and offering a stringent test of the model's generalization capability. As shown in Table 7, across both evaluation settings, the proposed DGDN model consistently outperforms all baselines in terms of Accuracy, Precision, Recall, and F1 Score. On the in-hospital split, DGDN achieves an F1 score of 85.6%, outperforming the NIH ChestX-ray14 baseline (83.9%) and MIMIC-CXR baseline (82.4%), with a relative improvement of +1.7% and +3.2% respectively. This suggests that even when evaluated on familiar institutional data, DGDN provides tangible gains through its multimodal integration and adaptive fusion mechanisms. More importantly, in the out-of-hospital split, which evaluates the model's robustness to unseen patient distributions and clinical protocols, DGDN maintains strong performance with an F1 score of 80.6%, clearly surpassing NIH ChestX-ray14 (77.7%), MIMIC-CXR (76.4%), and MIMIC-IV (74.1%). The performance gap between in- and out-of-hospital settings is also smallest for DGDN (5.0 percentage points), compared to 6.2% for NIH ChestX-ray14 and 8.3% for MIMIC-IV, confirming that DGDN exhibits superior generalization and lower overfitting risk in retrospective clinical contexts. These results directly validate the practical reliability of DGDN for real-world deployment. By demonstrating stable performance on retrospective datasets with different data sources, DGDN is shown to be more resilient to inter-institutional variation–an essential property for AI systems used in large-scale clinical environments. This evidence further supports the claim that our model is not merely overfitting benchmark datasets, but is capable of handling diverse, historically collected patient data with robustness and consistency.

**Table 7:** Retrospective validation of DGDN on MIMIC-CXR dataset.

**Model variant**	**In-hospital split (seen patients)**				**Out-of-hospital split (unseen patients)**			
	**Accuracy** ↑	**Precision** ↑	**Recall** ↑	**F1 score** ↑	**Accuracy** ↑	**Precision** ↑	**Recall** ↑	**F1 score** ↑
MIMIC-IV (43)	83.6 ± 0.3	81.4 ± 0.4	79.5 ± 0.3	80.4 ± 0.3	77.5 ± 0.3	75.2 ± 0.4	73.0 ± 0.3	74.1 ± 0.3
MIMIC-CXR (42)	85.1 ± 0.3	83.0 ± 0.4	81.8 ± 0.3	82.4 ± 0.3	79.2 ± 0.3	77.0 ± 0.4	75.8 ± 0.3	76.4 ± 0.3
NIH ChestX-ray14 (44)	86.5 ± 0.3	84.6 ± 0.3	83.2 ± 0.4	83.9 ± 0.3	80.4 ± 0.3	78.1 ± 0.3	77.3 ± 0.4	77.7 ± 0.3
**Ours (DGDN)**	**88.0** **±0.3**	**86.2** **±0.3**	**85.0** **±0.4**	**85.6** **±0.3**	**83.1** **±0.3**	**81.2** **±0.3**	**80.0** **±0.4**	**80.6** **±0.3**

Bold values indicate the best performance in each column.

## 5 Discussion

While the proposed DGDN framework was evaluated across diverse datasets to demonstrate its multimodal capabilities, we acknowledge that not all datasets reflect real-world endoscopic diagnostic scenarios. CMU-MOSEI and MIMIC-IV, though representative of conversational and structured clinical data respectively, are not inherently imaging-based nor collected in direct endoscopy contexts. Their inclusion in our study primarily serves to validate the model's cross-modal adaptability, rather than clinical integration in its current form. This distinction is crucial to interpret our findings accurately. The lack of unified datasets encompassing synchronized endoscopic images, patient dialogue, and structured EHR for the same individuals remains a barrier to comprehensive clinical validation. Future work should focus on building or accessing such integrated multimodal clinical datasets to enable end-to-end deployment and evaluation of systems like DGDN in practical gastroenterological workflows.

We acknowledge that the real-time clinical deployment of large language models (LLMs) remains technically challenging, particularly in high-speed procedural environments such as endoscopic surgery. The computational demands, latency, and infrastructure requirements of current LLM architectures limit their feasibility for synchronous interaction during procedures. In our proposed framework, LLMs are primarily intended to support near-real-time interaction outside of critical surgical loops–such as automated documentation, post-procedure summarization, and asynchronous clinical decision support. For example, LLMs can be used to generate structured endoscopy reports based on multimodal inputs (images, patient data, dialogue transcripts) shortly after the procedure, reducing physician documentation workload and improving consistency. Real-time intra-procedural guidance remains an aspirational goal, potentially realizable through future developments such as on-device LLM distillation, model compression, or hybrid cloud-edge deployments. Furthermore, a layered deployment strategy can be adopted, wherein lightweight decision rules or vision-language modules provide intra-operative cues, while full LLM-based synthesis is performed post-operatively. This hybrid paradigm balances responsiveness and computational tractability while preserving clinical utility.

Ethical considerations are paramount in the clinical application of AI models, particularly those involving sensitive patient data and automated diagnostic reasoning. Although this study utilizes publicly available de-identified datasets, real-world deployment would necessitate stringent adherence to privacy regulations such as HIPAA and GDPR. Furthermore, ensuring fairness across diverse patient populations is critical; AI systems must be evaluated for demographic biases that may arise from training data imbalance or institutional heterogeneity. Another concern is the explainability of model outputs. In high-stakes clinical settings, black-box predictions can undermine clinician trust and pose medico-legal challenges. Our framework addresses this partially through attention visualization and uncertainty quantification; however, further development of transparent reasoning mechanisms is essential. Future implementations should also account for informed consent regarding AI usage, clearly delineating the boundaries of machine-augmented recommendations vs. physician decision-making authority. Establishing oversight protocols, continuous auditing, and ethical review processes will be essential to safeguard patient safety, trust, and autonomy as AI tools like DGDN transition from research to clinical environments.

## 6 Conclusions and future work

The integration of large language models (LLMs) with multimodal learning presents a transformative opportunity in gastroenterology, particularly in digestive endoscopy. Traditional AI-assisted endoscopic systems primarily rely on single-modal image analysis, which lacks contextual awareness and adaptability to complex gastrointestinal (GI) conditions. These conventional approaches face critical limitations, such as domain shifts, data heterogeneity, and interpretability issues, which hinder their clinical applicability. To overcome these challenges, we propose a multimodal learning framework that seamlessly integrates LLM-powered chatbots with endoscopic imaging and patient-specific medical data. Our method leverages self-supervised learning to extract clinically relevant patterns from heterogeneous sources, enabling real-time guidance and AI-assisted report generation. A domain-adaptive learning strategy enhances model generalization across diverse patient populations and imaging conditions. Experimental evaluations on multiple GI datasets confirm that our approach improves lesion detection, reduces diagnostic variability, and enhances physician-AI collaboration, highlighting its potential to advance AI-driven gastroenterology.

Despite these promising results, our approach presents two primary limitations. Real-time processing efficiency remains a challenge due to the computational demands of multimodal data fusion and LLM inference. The integration of high-dimensional image data with LLM-based text processing requires substantial computational resources, which may limit deployment in resource-constrained clinical environments. Future research should focus on model optimization techniques, including quantization, pruning, and hardware acceleration, to improve efficiency. Model generalization across different medical institutions and populations requires further validation. While our domain-adaptive learning strategy mitigates some generalization issues, real-world variations in endoscopic equipment, clinical protocols, and patient demographics may introduce biases. Future work should explore continual learning and federated learning approaches to enhance adaptability while preserving patient privacy. Addressing these challenges will be essential for the successful integration of LLM-driven multimodal AI systems in digestive endoscopy, ultimately improving diagnostic accuracy, procedural efficiency, and clinical decision-making in gastroenterology.

## Data Availability

The original contributions presented in the study are included in the article/supplementary material, further inquiries can be directed to the corresponding author.
